# Ambient AI Scribes to Create Educational Feedback Notes for Medical Students: Randomized Trial

**DOI:** 10.2196/89996

**Published:** 2026-05-28

**Authors:** Jaideep S Talwalkar, David Chartash, Lisa Zhang, Michael Makutonin, Conrad W Safranek, Anne Elizabeth Sidamon-Eristoff, Lee H Schwamm, Donald S Wright

**Affiliations:** 1 Departments of Medicine and Pediatrics, Yale School of Medicine, 367 Cedar Street, Bldg D, New Haven, CT, 06510, United States, 1 203-737-4190, 1 203-737-4199; 2 Department of Emergency Medicine, Yale School of Medicine, New Haven, CT, United States; 3 Yale School of Medicine, New Haven, CT, United States; 4 Departments of Neurology and Biomedical Informatics and Data Sciences, Yale School of Medicine, New Haven, CT, United States; 5 Department of Veterans Affairs, Connecticut VA Healthcare System, West Haven, CT, United States

**Keywords:** ambient scribe, artificial intelligence, AI, feedback, medical student education, formative assessment, competency-based education

## Abstract

**Background:**

High-quality observation and feedback contribute to the development of clinical competence and professional growth in medical education. Faculty often struggle to translate verbal observations into written feedback because of documentation burden and competing demands. Ambient artificial intelligence (AI) scribes, already adopted in clinical practice, may address this challenge by capturing verbal exchanges and generating structured notes.

**Objective:**

The purpose of this study was to examine the use of ambient AI scribes to generate educational feedback notes during a formative medical interviewing workshop for first-year medical students in March and April 2025.

**Methods:**

Thirteen instructors were randomized to control (human-only) or intervention (AI scribe–assisted) workflows to complete narrative feedback forms. The intervention group used an AI scribe to generate transcripts of student-instructor encounters, which were then summarized into feedback notes using a large language model and edited by the instructors before submission. All narratives were scored using the Evaluation of Feedback Captured Tool (EFeCT). Factual accuracy of a subsample of unedited AI feedback summaries was reviewed against the source transcripts. Task load and usability were measured using NASA Task Load Index and System Usability Scale, respectively.

**Results:**

Instructors submitted feedback on 92.2% (94/102) of the students. EFeCT scores on the scale from 0 to 5 were higher for human-edited AI narratives (median 3.00, IQR 2.00-4.00) and unedited AI summaries (median 3.00, IQR 3.00-4.00) than for human-only narratives (median 2.00, IQR 1.75-3.00; *P*<.001). Human-only narratives were shorter than AI-assisted outputs (*P*<.001). Review of 117 AI-generated feedback elements showed a 6.8% (n=8) mischaracterization and 1.7% (n=2) hallucination rate, with most errors corrected during editing. Task load was high, and usability was marginal in both the control and intervention groups, with no significant differences (*P*=.31 and *P*=.40, respectively).

**Conclusions:**

An ambient AI scribe–assisted workflow improved the quality of written narrative feedback with no observed increase in instructor effort compared to human-only documentation. Although occasional inaccuracies required review, this innovation has the potential to transform feedback documentation.

## Introduction

### Problem: Documentation Burden and Quality of Feedback in the Modern Assessment Paradigm

High-quality observation and feedback contribute to the development of clinical competence and professional growth in medical education. Effective feedback provides learners with specific and actionable information on their performance, which can promote deliberate practice, motivation, and engagement with ongoing feedback [1-4]. While verbal feedback is critical for collaborative discussion [5] and occurs frequently in clinical [1] and simulation settings [6], it may be limited as a tool for longitudinal reflection as learners recall few feedback points when given verbal feedback alone [5,7]. Written feedback supports self-regulated learning by allowing trainees and their supervisors to review past narratives, set goals, and refine strategies for improvement [1,8]. By providing detailed, context-rich guidance, narrative feedback can help learners focus efforts on achieving professional outcomes expected of them during training [1,9].

In recognition of the value of both verbal and written feedback, accreditation bodies such as the Liaison Committee on Medical Education emphasize the importance of defined competencies within a robust assessment system that includes narrative feedback [10]. Unfortunately, medical educators faced with competing responsibilities and increased educational documentation burden [11] struggle to convert real-time verbal observations into meaningful written feedback [1]. As a result, rather than serving as a tool to promote learning, formative assessment is often reduced to a checklist activity disconnected from best practices for feedback. Brief, nonspecific narratives are common and diminish the educational value of written feedback [11,12].

### Solution: Ambient Artificial Intelligence Scribes in Educational Practice

Ambient artificial intelligence (AI) refers to AI embedded into environments, working continuously in the background to support human tasks [13]. Ambient AI scribes have seen rapid adoption to address the growing documentation burden in clinical practice. Ambient AI scribes passively capture and document physician-patient conversations into structured clinical notes [14]. There is early evidence suggesting that these tools reduce administrative burden while maintaining or improving documentation quality [15,16]. The potential for this technology to extend beyond patient care into medical education is compelling. By capturing and structuring verbal formative feedback provided by faculty in real time, ambient AI scribes could generate written records of learner performance. These “educational feedback notes” could transform narrative workflows similar to the way in which AI-generated clinical notes are transforming clinical practice [17].

### Gap: No Use in Medical Education Settings

Despite the promise of ambient AI scribes in clinical settings, their application in medical education remains unexplored. No studies have described the use of these tools in directly observed encounters or small-group teaching settings, examined whether they can reliably capture narrative feedback in teaching contexts, compared how their outputs align with best practices for written feedback, or determined whether faculty perceive them as usable while actively teaching. No extensions to summarize data into educational feedback notes currently exist. The potential for ambient AI scribes to transform feedback documentation in education remains untested.

This study is the first to our knowledge to examine the use of ambient AI scribes to generate educational feedback notes during directly observed standardized patient (SP) encounters. Specifically, we aimed to (1) evaluate the quality and accuracy of narrative feedback captured by an ambient AI scribe workflow compared to that of feedback provided by humans without AI assistance during a formative medical interviewing workshop for first-year medical students and (2) assess the usability of this technology by medical educators.

## Methods

### Educational Setting

This study took place in spring 2025 as part of a formative medical interviewing workshop for all first-year medical students at Yale School of Medicine. During the workshop, each medical student spent 20 minutes conducting a complete history with an SP while being observed by an instructor and 3 peers. Students and instructors were permitted to call “time-out” for interspersed guidance and feedback [6]. Upon completion of the interview, 10 minutes were allotted for self-, peer, and instructor feedback. SPs provided no feedback. The exercise was repeated until all 4 students in the room had interviewed separate SPs portraying different scenarios and received individual verbal feedback. After the workshop, instructors completed a postsession feedback narrative (Multimedia Appendix 1) for each student within a learning management system (Medtrics Lab LLC). The workshop was cycled over 4 afternoons, with each student participating once. Instructors devoted 3 and a half hours per workshop, including presession faculty development. This was the seventh iteration of the workshop in the first-year clinical skills curriculum, with each workshop structured similarly, offering students opportunities for repeat practice as their foundational knowledge grows during medical school.

### Participants and Platforms

Study participants were 13 clinician educators who had signed up to teach medical interviewing workshops based on their availability before the study was announced. All had completed standard training on teaching medical interviewing, facilitating small groups, working with SPs, and giving feedback. All had facilitated other medical interviewing workshops earlier in the academic year and completed feedback forms associated with those sessions. Instructors were informed of the study via email and in-person announcements.

All instructors received the same study-specific training on form completion and use of the verbatim transcription feature within the health system’s AI scribe (Abridge Inc), a generative AI tool based on a large language model (LLM) built using medical notes. The AI scribe transcripts were extracted separately from the final medical note that Abridge produces and then fed manually into a private instance of GPT-4o (OpenAI) within the university’s secure infrastructure (Clarity Platform; Multimedia Appendix 2) to produce a feedback summary that ignored the direct medical content and instead focused on student feedback. Other tools included a data collection instrument (Qualtrics International Inc) and the learning management system. Those wishing to opt out were instructed to inform a course administrator not involved in the study.

While the entire class of 104 medical students involved in the workshop was given the option to opt out, no students or instructors did.

### Feedback Workflows and Data Collection

**Figure 1.**
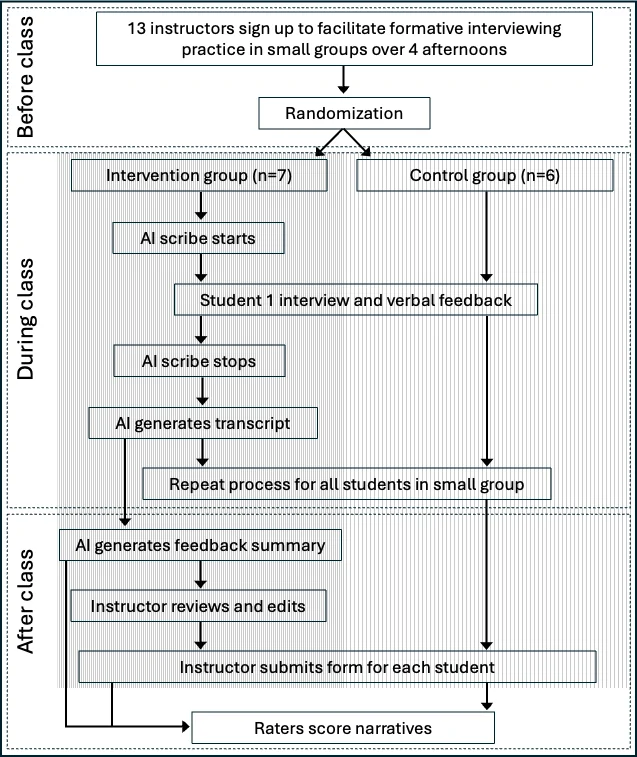
Depiction of the study workflow. Instructors were randomized to the control or intervention group; the latter used an artificial intelligence (AI) scribe to create a feedback summary. Three sources of narratives were analyzed: unedited AI, human-edited AI, and human only (control). Raters were blinded to the source of the narratives.

Instructors were randomized to the control or intervention group through random number generation to reduce bias of fixed effects from prior experience with the educational activity and technology, thereby isolating effects on the quality of the narratives (Figure 1). Instructors were informed of their group assignments immediately prior to the workshop. Students were unaware of their instructor’s group assignment, and all instructors placed their smartphones on a counter centrally located in the room at the start of the session. Control group instructors did not activate the AI scribe, and after completing the workshop, composed the “human narratives” on the postsession feedback forms without AI assistance. Intervention group instructors activated the AI scribe, which ran unobtrusively on a smartphone in the background, during each interview and feedback session to create a session transcript. After the workshop, they used a zero-shot prompt (ie, a prompting strategy in which the LLM is asked to perform a task without being given examples, training, or fine-tuning) [18,19] within the Clarity Platform (Multimedia Appendix 3) to extract the educational feedback comments and use them to create a feedback summary from each transcript. The prompt was written to summarize feedback verbalized during the educational session, not to create new feedback. Instructors reviewed and edited these outputs as they deemed necessary before entering them as final narratives, thus turning unedited AI feedback summaries into human-edited AI narratives.

All text created through this process was submitted via a data collection instrument designed to preserve student anonymity. The instrument also prompted instructors to complete the NASA Task Load Index [20] and System Usability Scale [21] to measure instructor cognitive load and general usability, respectively.

### Review of Narratives

Feedback narratives were assessed using the Evaluation of Feedback Captured Tool (EFeCT), a feedback quality scoring tool consisting of 5 elements, each of which represents an aspect of good written feedback. One point is given for each element present, for a total score of 0 to 5 [22]. Two blinded, independent raters (DC and LZ) assigned scores asynchronously for each narrative. Reconciliation of mental models for each element was performed after 10 and 25 narratives. After all narratives were scored, the 2 raters synchronously reconciled their scores into a final score for each element for each narrative using the constant comparative method. This final reconciled score was passed to a third rater (JST) to be confirmed. In addition to the use of EFeCT, the raters flagged whether they thought the narrative was generated through AI assistance (binary variable).

### Evaluation of Factuality of AI Feedback Summaries

Eight unedited AI feedback summaries were randomly sampled from the intervention cohort to benchmark any potential factual inaccuracies as an exploratory secondary analysis. These summaries were manually delimited into concepts by identifying each independent feedback statement that relied on an observed behavior in the simulation. Each individual feedback concept in the unedited AI feedback summary was cross-referenced with the full transcript of the session by an author (DSW) and classified for factuality (ie, whether the information in the written text corresponded to a real-world fact) [23]. Feedback was classified as correct if it matched the content of the original transcript, mischaracterized if it misrepresented the original context or meaning of the interaction in the transcript, or hallucinated if it referenced events not present in the transcript. Each feedback element classified as mischaracterized or hallucinated was then compared to the human-edited AI narrative to see whether the error was resolved.

### Statistical Analysis

Analyses were conducted in R (version 4.2.2; R Foundation for Statistical Computing). For the EFeCT dataset, total EFeCT feedback scores and word counts were compared across sources (human only, unedited AI, and human-edited AI) using Kruskal-Wallis tests, with pairwise Wilcoxon rank-sum tests with Holm correction for multiple comparisons. Individual EFeCT subscores (elements B-F) were treated as binary outcomes and compared across groups using the Fisher exact test and then pairwise when the omnibus test was significant with Holm correction. Factuality analysis was conducted using descriptive statistics. The Fisher exact test was used to compare completion rates. We tested assumptions of normality (Shapiro-Wilk) and homogeneity of variance (Levene) prior to the cognitive load and usability analysis, in which we used independent-sample 2-tailed *t* tests. All tests were 2 tailed with significance set at an α value of .05.

### Ethical Considerations

This study received an exemption from review by the Yale University Institutional Review Board due to its educational nature on January 23, 2025 (protocol ID 2000039478).

## Results

Each of the 13 instructors taught 1 to 4 afternoons of the 4-workshop cycle. Of the 104 students in the class, 2 (1.9%) were absent due to illness. Instructors submitted feedback forms via the data collection instrument on 92.2% (94/102) of the students. The 46.2% (6/13) of instructors randomized to the control group taught a mean of 1.7 (SD 0.5) sessions and submitted human-only narratives for 84.2% (32/38) of the students in their groups, all of which were submitted on the day of the session. The 53.8% (7/13) of instructors in the intervention group taught a mean of 2.6 (SD 1.1) sessions and submitted unedited AI feedback summaries for 95.3% (61/64) of the students and human-edited AI narratives for 96.9% (62/64) of the students, a completion rate significantly higher than that of the control group (*P*=.049). All were submitted within 1 week of the session, with 95.2% (59/62) submitted on the day of the session.

**Figure 2.**
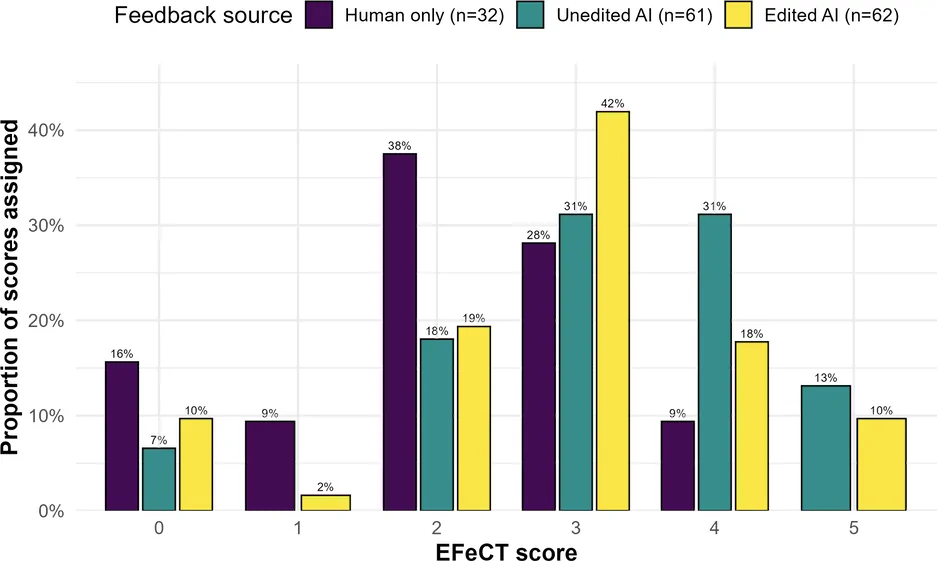
Evaluation of Feedback Captured Tool (EFeCT) scores by narrative source: human only, unedited artificial intelligence (AI), and edited AI. The EFeCT consists of 5 elements, each of which represents an aspect of good written feedback. One point is given for each element present for a total score of 0 to 5.

**Figure 3.**
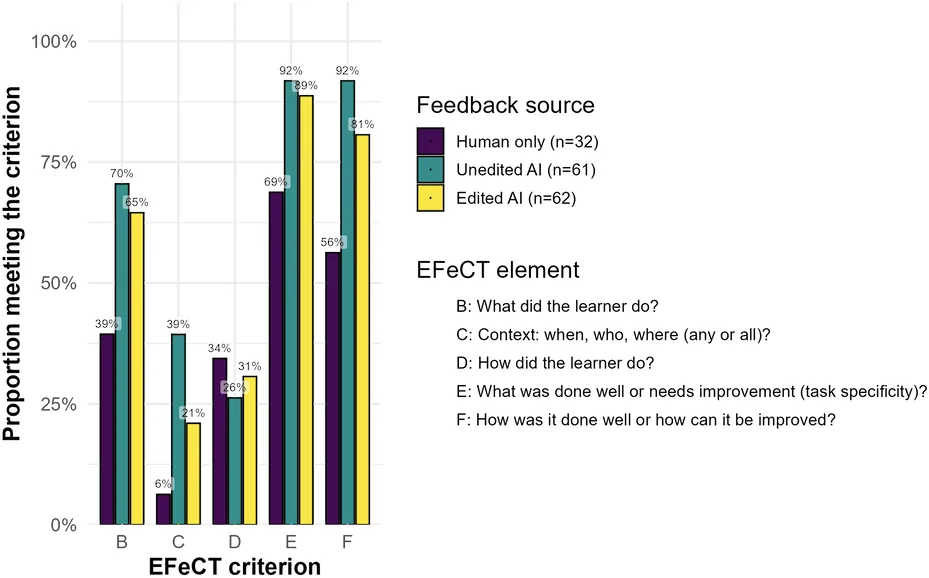
Evaluation of Feedback Captured Tool (EFeCT) scores by element. The EFeCT consists of 5 elements. One point is given for each element present. Element lettering begins with B because the “A” designation is used when the narrative section is blank, which did not apply to this study. AI: artificial intelligence.

Median EFeCT scores were 2.00 (IQR 1.75-3.00) for human-only narratives (control group), 3.00 (IQR 2.00-4.00) for human-edited AI narratives, and 3.00 (IQR 3.00-4.00) for unedited AI feedback summaries (Table 1). When narratives were grouped by feedback generation method, a significant difference in total EFeCT scores was observed (*P*<.001; Figure 2). Subsequent pairwise comparisons demonstrated higher EFeCT scores in both the human-edited and unedited AI feedback methods relative to the human-only narratives (*P*=.005 and *P*<.001, respectively), whereas no difference was observed between the 2 AI methods (*P*=.11). Similar significant overall and pairwise effects were observed on 4 of the 5 EFeCT component elements (Table 2 and Figure 3).

**Table 1.** Review of narratives, including factuality analysis.

Outcome	Narrative feedback workflow			Groupwise comparison	
	Human-only narratives (n=32)	Human-edited AI narratives^a^ (n=62)	Unedited AI narratives (n=61)	*P* value	Effect size estimate (ε^2^)
EFeCT^b^ score, median (IQR)	2.00 (1.75-3.00)	3.00 (2.00-4.00)	3.00 (3.00-4.00)	<.001^c^	0.11
Word count, median (IQR)	45.50 (33.00-62.00)	312.50 (207.25-355.00)	344.00 (310.00-396.00)	<.001^d^	0.40
Mischaracterizations among AI feedback elements, n (%)	—^e^	1/117 (0.9)	8/117 (6.8)	—	—
Hallucinations among AI feedback elements, n (%)	—	2/117 (1.7)	2/117 (1.7)	—	—

a AI: artificial intelligence.

b EFeCT: Evaluation of Feedback Captured Tool.

c Via the Kruskal-Wallis test. Additional pairwise comparisons demonstrated higher scores for edited (*P*=.005) and unedited (*P*<.001) AI narratives relative to human-only narratives.

d Additional pairwise comparisons demonstrated that all differences were statistically significant.

e Not applicable.

**Table 2.** Evaluation of Feedback Captured Tool (EFeCT) element comparisons.

Element and pairwise comparison	Pairwise *P* value^a^	Risk difference	Groupwise comparison^b^	
			*P* value	Effect size, Cramér *V*
What did the learner do? Interpretation: explicit statements that the participant performed a medical interview or completed a medical history.			.01	0.21
Unedited AI^c^ vs human-edited AI	.56	0.06		
Human only vs human-edited AI	.06	–0.25		
Human only vs unedited AI	.01	–0.31		
Context: when, who, where? Interpretation: a positive score was assigned if the raters were able to recognize which patient was being interviewed based on the context provided.			.001	0.27
Unedited AI vs human-edited AI	.06	0.18		
Human only vs human-edited AI	.08	–0.15		
Human only vs unedited AI	.002	–0.31		
How did the learner do? Interpretation: there was an explicit statement verbalized that the participant performed a medical interview AND a qualifier was provided stating how well they performed.			.70	0
Unedited AI vs human-edited AI	—^d^	—		
Human only vs human-edited AI	—	—		
Human only vs unedited AI	—	—		
What was done well or needs improvement? Interpretation: any explicit statement of feedback on a specific task.			.01	0.23
Unedited AI vs human-edited AI	.76	0.03		
Human only vs human-edited AI	.05	–0.20		
Human only vs unedited AI	.02	–0.23		
How was it done well or how can it be improved? Interpretation: any explicit statement that highlighted a specific element of a task that was done well or needed improvement.			<.001	0.31
Unedited AI vs human-edited AI	.11	0.11		
Human only vs human-edited AI	.03	–0.24		
Human only vs unedited AI	<.001	–0.36		

a Pairwise comparisons via the Fisher exact test with Holm correction for multiple comparisons; effect size reported as the risk difference (proportion meeting the criterion in group 1 minus group 2). A positive risk difference indicates higher EFeCT element attainment in the first-listed group.

b Via the Fisher exact test on 3 × 2 tables.

c AI: artificial intelligence.

d Not applicable.

Human-only narratives were significantly shorter (median 45.50, IQR 33.00-62.00 words) than those generated in human-edited (median 312.50, IQR 207.25-355.00 words) and unedited (median 344.00, IQR 310.00-396.00 words) AI feedback workflows (*P*<.001 in both cases). Human-edited AI narratives were shorter than unedited summaries (*P*=.001; Multimedia Appendix 4). A total of 117 AI-generated feedback elements were manually reviewed for factuality, demonstrating 8 (6.8%) mischaracterizations and 2 (1.7%) hallucinations in comparison to the source transcript. Most errors were corrected by instructors prior to submission of human-edited AI narratives (Table 1). Raters correctly identified 93.5% (58/62) of AI-assisted narratives and 100% (32/32) of human-only narratives.

Task load related to completion of the narratives as measured using the NASA Task Load Index did not differ between the control (mean 47.08, SD 12.39) and intervention (mean 54.32, SD 9.45) groups (*P*=.31; mean difference 95% CI –8.28 to 22.75), with scores indicating high cognitive load for both workflows [24]. Similarly, general usability as measured using the System Usability Scale did not differ between the control (mean 58.75, SD 19.55) and intervention (mean 49.70, SD 13.65) groups (*P*=.40; mean difference 95% CI –33.25 to 15.15), with scores indicating marginal acceptability [21].

## Discussion

### Quality of Narratives

The integration of an ambient AI scribe into educational workflows improved the quality of written feedback in a medical student workshop with no observed increase in instructor effort. Feedback narratives generated with AI assistance received significantly higher scores on the EFeCT instrument than those written by clinician educators without AI assistance. By passively capturing verbal feedback and restructuring it into written narratives, AI scribes may address a long-standing challenge in competency-based medical education: translating verbal observations into high-quality written narratives. This is the first study to our knowledge to explore the use of ambient AI scribes to create educational feedback notes, with improvement in documentation quality [15,16] and timeliness [25] similar to that observed with ambient scribes in clinical care.

The improvement observed with AI-assisted narratives is educationally meaningful: a 1-point increase on the EFeCT instrument exceeded the gains reported in the original validation study by Ross et al [22], where multiyear faculty development and feedback were required to achieve a smaller degree of improvement. Several factors may explain why AI-assisted narratives were scored higher on nearly all elements of the EFeCT instrument. Because the EFeCT instrument rewards inclusion of specific feedback elements, the longer AI-assisted narratives may have scored higher because they provided the detail needed to explicitly include those elements. The zero-shot LLM, guided only by a simple prompt, drew on a broad knowledge base of feedback principles, generating more structured, context-rich narratives. In contrast, despite prior extensive training in delivering verbal and written feedback, instructor-written narratives omitted best practice suggestions, as has been described previously. In an integrative review on feedback practices, Bing-You et al [26] identified inadequate feedback as a frequent theme in the literature even among experienced educators and those who had received feedback training. Gingerich et al [12] highlight a tendency for faculty to either omit written feedback or provide only vague, nonspecific comments, particularly when they feel that essential feedback has already been delivered verbally or is considered “unwritable” due to social or relational dynamics. Additionally, instructors may have relied on the assumption that students will sufficiently retain verbal feedback given during the encounter despite evidence to the contrary [7]. Finally, despite having volunteered, instructors may have faced competing professional demands that accumulated during the 3.5-hour workshop, potentially limiting time spent on feedback forms [1,11].

The impact of an AI-assisted workflow in narrative feedback may be even greater outside of research settings. In this study, all instructors were clinician educators who were aware that their notes would be reviewed as part of the research protocol. This may have resulted in more detailed narratives from instructors in the control group than they would have submitted otherwise. Additionally, nearly all narratives were submitted immediately. In real-world educational environments, where delays in form completion are common and specificity is often lost due to poor recall [27], the potential benefit of ambient AI scribes may be even greater. Alternatively, observed differences in completion rates may be attributable to novelty of the intervention workflow, warranting iterative evaluation of sustained impact.

### Accuracy and Reliability of AI Outputs

As a secondary, exploratory outcome, we also attempted to measure the rate of hallucinations and mischaracterizations. Within the sample of unedited feedback summaries that were examined, factual inconsistencies with the underlying transcript were rare; however, the certainty of this estimate is limited by the size of the sample reviewed. Instructors corrected most of the errors in the unedited AI feedback summaries, resulting in shorter human-edited AI narratives that retained higher EFeCT scores. This finding underscores the importance of human oversight over AI outputs. Accurate documentation in medical education remains paramount given its importance for learner development, institutional accountability, and public trust in training outcomes [9].

### Task Load and Usability

We did not observe reductions in task load or improvements in usability when using AI scribes compared to the traditional manual human workflow. Both approaches were rated as cognitively demanding and only marginally usable. Notably, the manual workflow itself was associated with high task load, echoing prior work showing that educational documentation can add extraneous demands that detract from core teaching activities [1,11,28]. Cognitive load theory dictates that, when administrative processes create high extraneous load, educators have fewer cognitive resources available for essential instructional and clinical tasks [29]. This dynamic likely contributes to the tendency of faculty to abbreviate written feedback despite their awareness of best practices.

Our findings contrast with reports from clinical medicine, where ambient AI scribes have consistently reduced cognitive burden and improved usability for clinicians [14,16,30,31]. The discrepancy likely reflects the research workflow we used, which required manual copying and pasting among 3 separate platforms (Abridge, Clarity, and Medtrics), all of which were used at no added cost as they were already available within the university and health system. By design, the copy-paste workflow introduced inefficiencies to capture interim outputs for study purposes that would be unnecessary in practice. In a production-grade system, this would be replaced by automated data flow, which may lead to better task load and usability relative to our intervention, so our findings related to cognitive load and usability should be interpreted with caution. Additionally, with well-designed systems, cognitive load [29] and usability [21] typically improve with experience, suggesting that adoption over time may mitigate some of the present challenges as workflows become smoother and more familiar.

### Limitations

This study has several limitations. It was conducted at a single institution with a small number of highly trained educators, limiting generalizability. Participants were diligent in reviewing AI outputs, which may not reflect the behaviors of a broader faculty population in usual teaching environments. However, in real-world practice, we can reasonably expect human-only narratives to be less diligently submitted, potentially further increasing the observed differences in quality between AI-assisted and human-only narratives.

Additionally, the AI workflow relied on robust verbal feedback having occurred as the scribe was not enabled to generate its own feedback. We used this safeguard to ensure that feedback was consistent with session objectives.

Second, some contamination may have occurred if instructors in the intervention arm occasionally defaulted to human-only narratives, potentially due to usability challenges. Raters were excellent at identifying AI-assisted narratives, so the rare occasions when raters misattributed an AI-generated narrative to the human-only group raise the possibility that technical difficulties or workflow barriers led some intervention instructors to manually compose narratives. If this occurred, the observed differences in quality between AI-assisted and human-only narratives may, in fact, underestimate the true effect of AI assistance.

Finally, while the EFeCT instrument provides a framework for evaluating written feedback, this was its first application to our knowledge to AI-generated narratives. The EFeCT instrument focuses on structure and content but does not capture tone or learner-perceived utility.

### Future Directions

Next steps should expand on these findings in several directions. It is essential to engage learners and clinical coaches to determine whether AI-assisted narratives are useful or actionable for reflection and growth. Through prompt engineering, AI outputs can be iteratively refined to more closely align with the needs of learners and coaches and adjusted to improve factuality [32]. Additionally, integrating AI scribe content into streamlined workflows and data pipelines could reduce task load and facilitate adoption. Finally, applications in other educational settings—such as clinical precepting and bedside teaching—represent promising areas where ambient AI could help close the gap between frequent verbal feedback and inadequate written documentation.

### Conclusions

Ambient AI scribes represent a promising innovation in medical education, where the challenge of creating high-quality narrative feedback persists. In this study, AI-assisted feedback narratives—produced through the combination of automated transcription and zero-shot prompting of an LLM—were of higher quality than human-only narratives with no observed increased human effort. While usability gains were not realized in this research workflow, integration into streamlined educational systems could replicate the successes of ambient AI scribes observed in clinical practice. With continued refinement and oversight, ambient AI scribes have the potential to strengthen feedback culture; support longitudinal assessment; and, ultimately, enhance the learning experience for medical students.

## Supplementary material

10.2196/89996 Multimedia Appendix 1 Postsession form completed by the instructor for each student. This standard formative assessment form is used across curricular sessions during which medical students are directly observed performing the patient care skills of history taking or physical examination. Instructors compose a feedback narrative in the final open-response box.

10.2196/89996 Multimedia Appendix 2 Clarity Platform technical specifications.

10.2196/89996 Multimedia Appendix 3 Zero-shot prompt in GPT-4o used by the intervention group.

10.2196/89996 Multimedia Appendix 4 Sample feedback notes submitted by instructors to first-year medical students following the formative medical interviewing workshop. Three representative samples from each workflow are presented with the removal of student identifiers. Control group instructors created the narratives manually. Intervention group instructors created the narratives with the use of an ambient artificial intelligence scribe.

## Abbreviations

AI: artificial intelligence
EFeCT: Evaluation of Feedback Captured Tool
LLM: large language model
SP: standardized patient

## References

[1] Cooper D, Holmboe ES (2025). Competency-based medical education at the front lines of patient care. N Engl J Med.

[2] Watling CJ, Ginsburg S (2019). Assessment, feedback and the alchemy of learning. Med Educ.

[3] Veloski J, Boex JR, Grasberger MJ, Evans A, Wolfson DB (2006). Systematic review of the literature on assessment, feedback and physicians’ clinical performance: BEME Guide No. 7. Med Teach.

[4] Lai MM, Roberts N, Mohebbi M, Martin J (2020). A randomised controlled trial of feedback to improve patient satisfaction and consultation skills in medical students. BMC Med Educ.

[5] Natesan S, Jordan J, Sheng A (2023). Feedback in medical education: an evidence-based guide to best practices from the Council of Residency Directors in Emergency Medicine. West J Emerg Med.

[6] Talwalkar JS, Cyrus KD, Fortin AH (2020). Twelve tips for running an effective session with standardized patients. Med Teach.

[7] Humphrey-Murto S, Mihok M, Pugh D, Touchie C, Halman S, Wood TJ (2016). Feedback in the OSCE: what do residents remember?. Teach Learn Med.

[8] Sargeant JM, Mann KV, van der Vleuten CP, Metsemakers JF (2009). Reflection: a link between receiving and using assessment feedback. Adv Health Sci Educ Theory Pract.

[9] Holmboe ES, Sherbino J, Long DM, Swing SR, Frank JR (2010). The role of assessment in competency-based medical education. Med Teach.

[10] Publications. Liaison Committee on Medical Education.

[11] Szulewski A, Braund H, Dagnone DJ (2023). The assessment burden in competency-based medical education: how programs are adapting. Acad Med.

[12] Gingerich AN, Lingard L, Sebok-Syer SS, Watling CJ, Ginsburg S (2024). “Praise in public; criticize in private”: unwritable assessment comments and the performance information that resists being written. Acad Med.

[13] Tierney AA, Gayre G, Hoberman B (2025). Ambient artificial intelligence scribes: learnings after 1 year and over 2.5 million uses. NEJM Catalyst.

[14] Gams M, Gu IYH, Härmä A, Muñoz A, Tam V (2019). Artificial intelligence and ambient intelligence. J Ambient Intell Smart Environ.

[15] Cain CH, Davis AC, Broder B (2025). Quality assurance during the rapid implementation of an AI-assisted clinical documentation support tool. NEJM AI.

[16] Tierney AA, Gayre G, Hoberman B (2024). Ambient artificial intelligence scribes to alleviate the burden of clinical documentation. NEJM Catalyst.

[17] Fu P, Zheng K, Westbrook J, Patel VL (2025). Reengineering Clinical Workflow in the Digital and AI Era.

[18] Liu J, Liu F, Wang C, Liu S (2025). Prompt engineering in clinical practice: tutorial for clinicians. J Med Internet Res.

[19] Huo B, Collins GS, CHART Collaborative (2025). Reporting guideline for chatbot health advice studies: the CHART statement. JAMA Netw Open.

[20] Hart SG (2006). NASA-Task Load Index (NASA-TLX); 20 years later. Proc Hum Factors Ergon Soc Annu Meet.

[21] Bangor A, Kortum PT, Miller JT (2008). An empirical evaluation of the System Usability Scale. Int J Hum Comput Interact.

[22] Ross S, Hamza D, Zulla R, Stasiuk S, Nichols D (2022). Development of and preliminary validity evidence for the EFeCT feedback scoring tool. J Grad Med Educ.

[23] Saurí R, Pustejovsky J (2012). Are you sure that this happened? Assessing the factuality degree of events in text. Comput Linguist.

[24] Prabaswari AD, Basumerda C, Utomo BW (2019). The mental workload analysis of staff in study program of private educational organization. IOP Conf Ser Mater Sci Eng.

[25] Moura LM, Mishuris RG, Metlay JP (2026). Hybrid ambient clinical documentation and physician performance: work outside of work, documentation delay, and financial productivity. J Gen Intern Med.

[26] Bing-You R, Varaklis K, Hayes V, Trowbridge R, Kemp H, McKelvy D (2018). The feedback tango: an integrative review and analysis of the content of the teacher-learner feedback exchange. Acad Med.

[27] Lee GB, Chiu AM (2022). Assessment and feedback methods in competency-based medical education. Ann Allergy Asthma Immunol.

[28] Li SX, Li CMF, Jenkins ME, Venance SL, Florendo-Cumbermack A (2023). Insights from the transition to competency-based medical education in neurology programs. Can J Neurol Sci.

[29] Young JQ, Van Merrienboer J, Durning S, Ten Cate O (2014). Cognitive load theory: implications for medical education: AMEE guide no. 86. Med Teach.

[30] Wright DS, Kanaparthy NS, Melnick ER (2025). The effect of ambient artificial intelligence scribes on trainee documentation burden. Appl Clin Inform.

[31] Olson KD, Meeker D, Troup M (2025). Use of ambient AI scribes to reduce administrative burden and professional burnout. JAMA Netw Open.

[32] Debnath T, Siddiky MN, Rahman ME (2025). A comprehensive survey of prompt engineering techniques in large language models. TechRxiv.

