# Supplementary figures and images for: Ambient AI Scribes to Create Educational Feedback Notes for Medical Students: Randomized Trial
Source: JMIR Med Educ. 2026 May 28;12:e89996. doi: 10.2196/89996 (PMC13218648; doi:10.2196/89996)

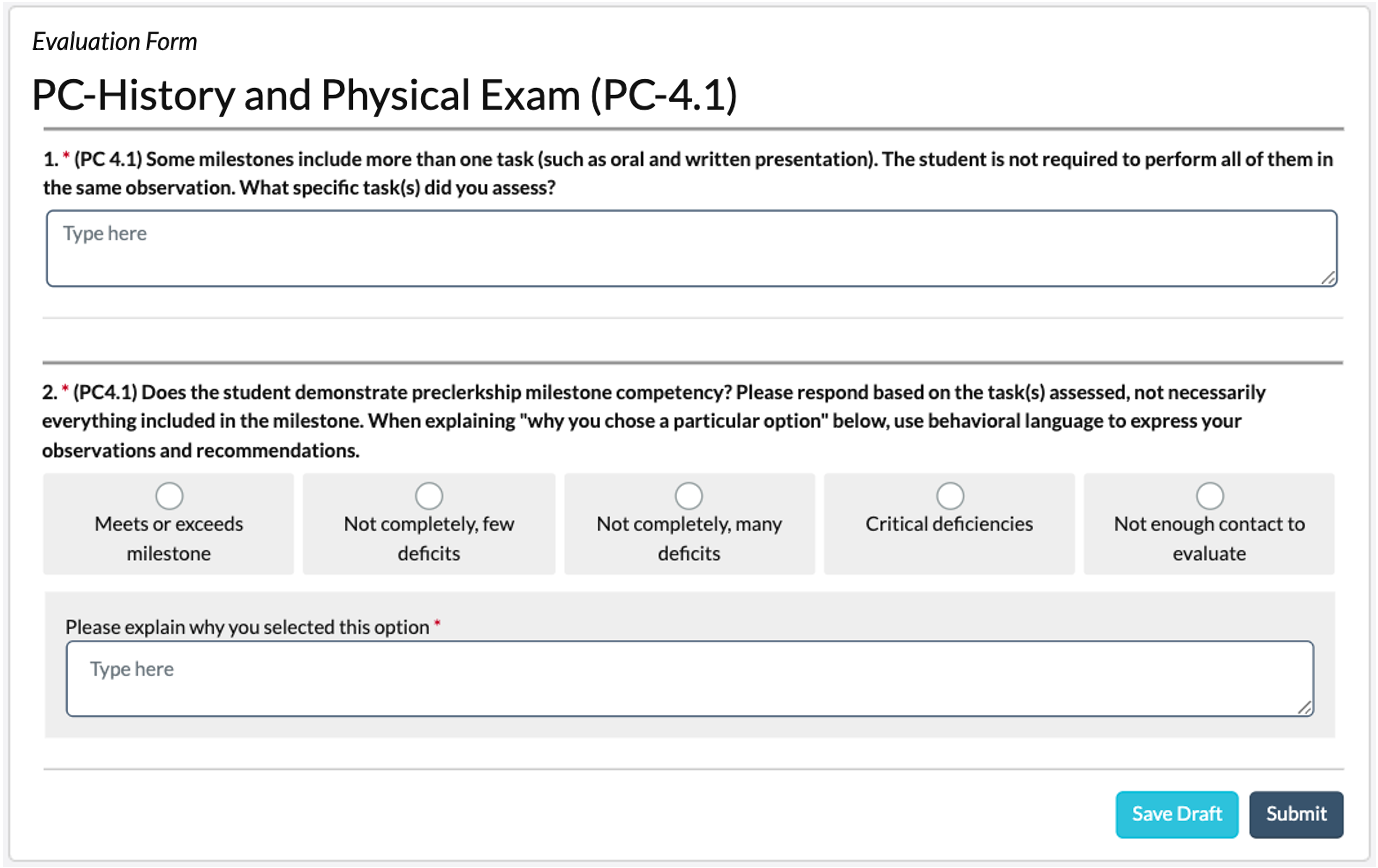

Supplement: Multimedia Appendix 1 [file mededu-v12-e89996-s001.png]
