# Supplementary material for: Ambient AI Scribes to Create Educational Feedback Notes for Medical Students: Randomized Trial
Source: JMIR Med Educ. 2026 May 28;12:e89996. doi: 10.2196/89996 (PMC13218648; doi:10.2196/89996)
Supplement: Multimedia Appendix 2 [file mededu-v12-e89996-s002.docx]

Appendix 2. Clarity Platform technical specifications

The Clarity Platform is a generative AI tool housed within the university’s secure infrastructure. Various models are available within the Clarity Platform. For this study, we used the following technical specifications within the Clarity Platform.

Model name: GPT-4o

Release date: May 13, 2024

GPT-4o is a closed-source/proprietary, tuned model developed by OpenAI. Its architecture and training data are not publicly released in full detail. It is optimized for real-time multimodal interactions across text, audio, and vision. GPT-4o was tuned using supervised fine-tuning and reinforcement learning from human feedback (RLHF). It was trained on a mixture of publicly available and licensed datasets. Specific datasets and tuning parameters have not been disclosed by OpenAI.
