# Supplementary material for: Ambient AI Scribes to Create Educational Feedback Notes for Medical Students: Randomized Trial
Source: JMIR Med Educ. 2026 May 28;12:e89996. doi: 10.2196/89996 (PMC13218648; doi:10.2196/89996)
Supplement: Multimedia Appendix 3 [file mededu-v12-e89996-s003.docx]

Appendix 3. Zero-shot prompt in GPT-4o used by intervention group

This document is a transcript of a medical student who just interviewed a patient while periodically receiving feedback from peers and a medical school professor who specializes in medical education. Please extract the feedback provided to the student during the session, which can then be given to the student for future improvement.
