# Supplementary material for: Ambient AI Scribes to Create Educational Feedback Notes for Medical Students: Randomized Trial
Source: JMIR Med Educ. 2026 May 28;12:e89996. doi: 10.2196/89996 (PMC13218648; doi:10.2196/89996)
Supplement: Multimedia Appendix 4 [file mededu-v12-e89996-s004.docx]

Appendix 4. Sample feedback notes submitted by instructors to first year medical students following formative medical interviewing workshop. Three representative samples from each workflow are presented here with removal of student identifiers. Control group instructors created narratives manually. Intervention group instructors created narratives with the use of an ambient artificial intelligence scribe.

Control group sample feedback notes.

Sample from instructor 1

Good empathy and use of pain scale. Used both open and closed questions and good summary!

Sample from instructor 2

The student did a great job with the interview today. He was clear, efficient, and compassionate. He did a very nice job with the case. We discussed fleshing out NURS statements a bit more.

Sample from instructor 3

Great job setting the scene and exhausting the agenda ("overview of visit"). Fantastic job using gentle approach to a sensitive topic in a defensive patient (Asked for permission, reflecting verbatim, and several NURS statements). Could consider asking about previous treatments, complications, and medical teams for more chronic chief complaints.

Intervention group sample feedback notes.

Sample from instructor 4

Here's a summary of the feedback provided to the medical student during the session, which can be useful for future improvement:

Positive Feedback:
1. Redirecting Focus Appropriately:
 - The student successfully redirected the focus of the interview from a possible physical trauma to understanding the social and mental health aspects contributing to the patient's condition.

2. Allowing the Patient to Tell Their Story:
 - The student allowed the patient to fill in the blanks and guide the conversation, facilitating a comprehensive understanding.

3. Empathy and Normalizing Statements:
 - The student made effective use of normalizing statements, helping the patient feel comfortable and less ashamed about discussing sensitive topics like food-related issues and purging.

4. Effective Introduction and Conclusion:
 - The student did a good job at the beginning by introducing herself and explaining her role and at the end by summarizing the next steps, so the patient had clear expectations.

5. Building Comfort and Trust:
 - The patient felt comfortable enough to disclose details about laxative use and purging patterns, indicating that the student created a trusting environment during the interview.

Feedback for Improvement:
1. Timing and Efficiency:
 - The student expressed the need to improve on timing, particularly finding a balance in how to navigate and prioritize social history within the limited time frame. Learning to strategize within the time constraints of a full medical interview is essential.

2. Avoiding Double-Barreled Questions:
 - It is important to ask about each substance (alcohol, tobacco, and drugs) individually rather than in clusters to avoid confusion and ensure accurate responses.

3. Specificity in Screening Questions:
 - The student should ask specific, individualized questions regarding immunizations and other health screenings rather than generalizing the questions to ensure precise and comprehensive information.

4. Memorizing Key Components:
 - The student identified the need to memorize the structure and key components of the interview, particularly the review of systems, to avoid reliance on notes and enhance efficiency and fluidity during the patient encounter.

5. Navigating Sensitive Topics:
 - The student should continue to develop skills in sensitively approaching and navigating potentially stigmatizing issues such as eating disorders, using the patient’s terminology and leading them to the required level of detail.

Overall, the student demonstrated effective communication, empathy, and appropriate redirection during the patient interview, with some areas identified for future improvement in timing, questioning technique, and navigating sensitive topics effectively.

Sample from instructor 5

Here is the summarized feedback provided to the student during the session for future improvement:

Positive Aspects:

Comforting Presence: The student had a very comforting presence, especially when the patient shared sensitive information about taking codeine. The student reassured the patient and thanked her for sharing, which helped in developing rapport and made the patient more comfortable.

Empathy and Acknowledgement: The student demonstrated good empathy, particularly when the patient talked about leaving her work and the challenges faced. The student acknowledged the patient's hardships and showed understanding.

Reassurance: The student did well in early responses, reassuring the patient about the importance of her visit and showing appreciation for her efforts to seek medical help.

Areas for Improvement:

Open-Ended Questions: At the beginning, because of nerves, the student moved on from open-ended questions quicker than desired. Next time, the student should try to leave more space for these questions to gather more complete patient narratives.

Focused Differential Diagnosis: The student recognized that at one point, she was getting invested in the patient's story rather than thinking about differential diagnosis. It's important to balance empathy with clinical reasoning to gather all relevant information.

Question Wording: There was a suggestion to avoid using the term “recreational drugs” as it may not encompass the intent behind non-prescribed drug use. Instead, asking if the patient uses any non-prescribed substances or drugs provides a clearer and less assumptive approach.

Specific Symptom Questions: More focused questions about the heel could have been asked, such as whether it was red, swollen, if there were any skin changes, if pressing on it caused pain, or if the pain could be recreated by certain movements.

Peer and Professor Feedback:

Empathic Communication: Peers appreciated the student’s empathetic statements and how she acknowledged the patient’s transitions and difficulties.

Use of Frameworks: The professor suggested using a framework for conditions like heel pain to ensure all relevant questions are covered in a structured way. Physicians in clinical settings often have frameworks in their mind to help structure interviews and ensure no significant differential diagnosis is overlooked.

This feedback can guide the student towards refining her clinical interview skills and improving patient interactions.

Sample from instructor 6

The feedback provided to you for future improvement includes several key points:

Positive Remarks:

Welcoming Statement: You did well in making patients feel comfortable right from the start by asking if there's anything you can do to make them feel comfortable.

Supportive Statements: You used supportive statements effectively, such as "I'm glad you came in today" and asking "What do you think contributes to your stress?" These helped build rapport and ease the patient's concerns.

Empathy and Personal Connection: You took the time to talk to the patient as a person, such as asking about her daughter and her activities. This shows that you care about her life beyond her medical concerns.

Bright and Warm Presence: Your natural brightness and warmth made a positive difference in the patient experience.

Constructive Feedback:

Getting Back into the Groove: You acknowledged that sometimes you knew what you wanted to say but had trouble expressing it. You'll become more fluent and confident in your interactions with more practice.

Prioritizing Concerns: You did well focusing on the primary concern and then addressing secondary issues if time allowed. This is important when managing limited time with patients.

Suggestions for Improvement:

Substituting Filler Words: Instead of saying "great" or "sounds good," try substituting these with more attentive listening cues like "I get it" or "Got it."

Avoiding Diminishing Language: Instead of saying "tell me a little bit about it," ask the patient to "tell me all about it" to encourage a more thorough response.

Agenda Setting: When there are multiple issues, explicitly ask the patient about their priority. This ensures that the most pressing concern is addressed first, and you can communicate this effectively to the attending or primary care provider.

Overall, the feedback highlighted areas where you excel and provided guidance on improving your patient interactions.
